# Genomic Deletion of *BAP1* and *CDKN2A* Are Useful Markers for Quality Control of Malignant Pleural Mesothelioma (MPM) Primary Cultures

**DOI:** 10.3390/ijms19103056

**Published:** 2018-10-07

**Authors:** Kadir Harun Sarun, Kenneth Lee, Marissa Williams, Casey Maree Wright, Candice Julie Clarke, Ngan Ching Cheng, Ken Takahashi, Yuen Yee Cheng

**Affiliations:** 1 Asbestos Diseases Research Institute, University of Sydney, Sydney, NSW 2139, Australia; kadir.sarun@sydney.edu.au (K.H.S.); kenneth.Lee@health.nsw.gov.au (K.L.); marissa.williams@sydney.edu.au (M.W.); cmdodds84@gmail.com (C.M.W.); ken.takahashi@sydney.edu.au (K.T.); 2 Anatomical Pathology Department, Concord Repatriation General Hospital, Sydney, NSW 2139, Australia; Candice.Clarke@health.nsw.gov.au; 3 School of Medicine, University of Sydney, Sydney, NSW 2006, Australia; 4 Liver Injury and Cancer Program, Centenary Institute, Sydney, NSW 2050, Australia; ngan.cheng@gmail.com

**Keywords:** mesothelioma, biomarker, FISH, genomic deletion, copy number variation, ddPCR

## Abstract

Malignant pleural mesothelioma (MPM) is a deadly cancer that is caused by asbestos exposure and that has limited treatment options. The current standard of MPM diagnosis requires the testing of multiple immunohistochemical (IHC) markers on formalin-fixed paraffin-embedded tissue to differentiate MPM from other lung malignancies. To date, no single biomarker exists for definitive diagnosis of MPM due to the lack of specificity and sensitivity; therefore, there is ongoing research and development in order to identify alternative biomarkers for this purpose. In this study, we utilized primary MPM cell lines and tested the expression of clinically used biomarker panels, including CK8/18, Calretinin, CK 5/6, CD141, HBME-1, WT-1, D2-40, EMA, CEA, TAG72, BG8, CD15, TTF-1, BAP1, and Ber-Ep4. The genomic alteration of *CDNK2A* and *BAP1* is common in MPM and has potential diagnostic value. Changes in *CDKN2A* and *BAP1* genomic expression were confirmed in MPM samples in the current study using Fluorescence In situ Hybridization (FISH) analysis or copy number variation (CNV) analysis with digital droplet PCR (ddPCR). To determine whether MPM tissue and cell lines were comparable in terms of molecular alterations, IHC marker expression was analyzed in both sample types. The percentage of MPM biomarker levels showed variation between original tissue and matched cells established in culture. Genomic deletions of *BAP1* and *CDKN2A*, however, showed consistent levels between the two. The data from this study suggest that genomic deletion analysis may provide more accurate biomarker options for MPM diagnosis.

## 1. Introduction

Malignant pleural mesothelioma (MPM) is a tumor originating from the mesothelium, the membrane lining the thoracic and peritoneal cavities [1]. MPM is strongly linked to previous asbestos exposure [2] and asbestiform minerals such as erionite and fluoroedenite [3]. Australia has one of the world’s highest incidences of MPM due to the heavy industrial utilization of asbestos in the past [4]. MPM is a deadly cancer with poor prognosis [1,5,6], and treatment options are mainly palliative [7]. Most MPM patients are diagnosed at a late stage of the disease where limited treatment options are available; this is due to a lack of symptoms at early stages and the long latency period between asbestos exposure and the development of MPM. Mesothelioma is especially difficult to diagnose, as symptoms closely resemble those of lung cancer. Delays or errors in diagnosis hinder treatment intervention that can subsequently adversely affect the patients’ survival and quality-of-life (QoL); therefore, accurate diagnosis is essential for prognostic and therapeutic purposes [8].

Immunohistochemistry (IHC) is the standard method for biomarker detection of MPM, and multiple mesothelial markers have been identified to enable the distinction between epithelioid MPM and adenocarcinomas in routine practice. The three predominant subtypes differentiated by their MPM histomorphology are epithelioid, biphasic, and sarcomatoid. The proteins assessed using IHC vary in different laboratories, but the use of antibodies for the identification of calretinin and CEA is prominent [8]. To date, it is generally accepted that no single biomarker is absolutely sensitive or specific for MPM, and multipanel immunohistochemical tests are essential for diagnosis [9,10]. Therefore, further molecular characterization of the tumor is required to potentially identify more specific markers to aid in the diagnosis of MPM.

As well as intertumor heterogeneity, MPM tumors also exhibit intratumor heterogeneity. This tumour complexity limits the ability to delegate suitable treatment options due to the existence of several tumor clones and subclones within a single patient [11]. Intertumor heterogeneity is inclusive of the variable molecular phenotype of MPM. While genomic loss and gain are evident in MPM tumors, they exhibit low levels of drivers and recurrent mutations in comparison to other cancers [12]. The most commonly reported mutations are identified in genes such as *NF2*, *BAP1*, *TP53*, *NRAS*, and *EGFR* [13,14]. Asbestos fibers have been demonstrated to induce chromosome instability resulting in dysfunctional DNA damage response [15]. The most frequently reported chromosomal losses are those affecting chromosomal arms 3p, 9p, and 22q. Genes located in these regions include *BAP1*, *CDKN2A*, and *NF2*, respectively [16,17]. Among the three, *CDKN2A* represents the highest number of homozygous deletions in MPM-patient tumors [18]. To date, the mechanisms underlying the poor response of MPM patients to a wide range of therapeutic interventions are largely undetermined. Molecular intertumor heterogeneity, including a diversity of mutation, epigenetic, expression, and microscopic (phenotypic) changes may cause inefficacy of the treatment regimens. In contrast to nonsmall cell lung cancer, many mutations, such as in *EGFR* or *TP53*, are uncommon in the majority of MPM cases [9,19].

Due to the lack of single, accurate biomarkers for MPM, recent studies have focused on the analysis of biomarker combinations or panels, as well as the development of new diagnostic methods separate from IHC. For example, the determination of *p16* (*CDKN2A*) homozygous deletion using Fluorescence In Situ Hybridization (FISH) and identification of BRCA1-associated protein 1 (BAP1) loss by IHC are particularly useful to differentiate mesothelial hyperplasia (MH) from MPM. Currently, these two markers are not widely used in the clinic, potentially due to the low sensitivity of the existing detection method [20,21,22,23]. Considering this, new markers with increased sensitivity and specificity are thus required. Cell-line models derived from MPM tumors are useful for the discovery of biomarkers and testing their efficacy. Cell culture is limitlessly renewable and can be manipulated to study cell function and gene signatures. Cultured cells derived from tumors have been shown to maintain many of the hallmarks of cancer apart from tumor-specific angiogenesis [24,25]. The development of a primary MPM cell culture provides an inexpensive and more homogeneous MPM cell population for genomic marker identification. Primary MPM cell lines have provided a medium to better understand the genomic alterations that exist in mesothelioma [26]. Further, in tumor samples, the inevitable infiltration of stromal and inflammatory cell populations can influence the molecular phenotype. The implementation of cell-culture models allows the exclusion of such populations, and allows exclusive testing of the tumor cells and accurate estimation of gene copy number.

This study aimed to utilize the MPM cell lines that were established between 2013–2017 [27] to study different types of biomarkers, including protein markers using IHC and genomic markers using qualitative FISH analysis coupled with absolute quantification analysis with droplet digital PCR (ddPCR).

## 2. Results

### 2.1. Immunohistochemistry Analysis Demonstrates Variable Marker Expression between MPM Tissue and Derivative Cell-Line Samples

**Figure 1:**
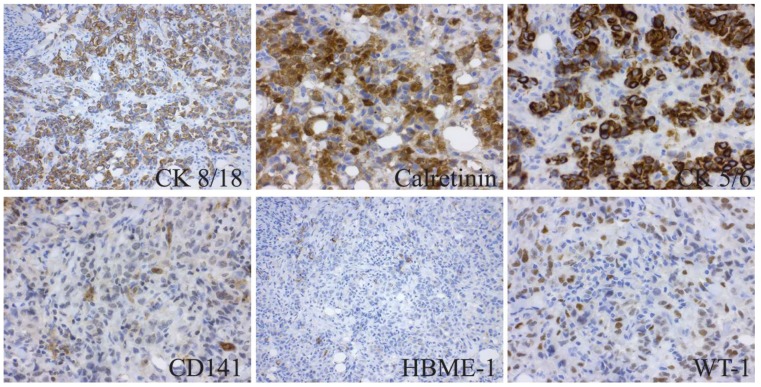

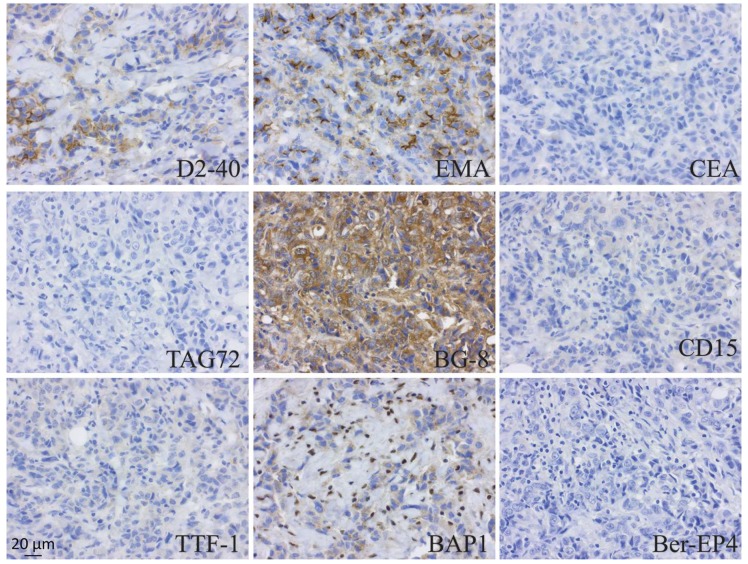
Representative immunohistochemical (IHC) staining of a malignant pleural mesothelioma (MPM) (sample ID 1157) patient tumor sample with the 15 biomarkers currently used for clinical diagnosis. *BAP1* is not expressed in sample MM ID 1157, therefore *BAP1* staining of sample MM ID 1518 is included as an example of positive *BAP1* expression in this figure. All pictures are taken at same magnification with scale bar indicated left bottom corner of TFF-1 staining.

A total of 15 biomarkers used in clinical practices for differential diagnosis of MPM were assessed in all samples, including MPM tumor samples together with derivative primary cell-line samples from the corresponding parent tumor tissue and/or plural effusion. Short Tandem Repeat (STR) profiling was employed to provide genomic signatures for confirmation of cell-line identity (Supplementary Table S1). Figure 1 shows the IHC staining of a MPM tumor and the expression of 15 protein markers used in the clinic. MPM primary cells were extracted from tumor tissue and/or pleural effusion samples and grown in cell culture until they reached passage 15 to eliminate normal cell contamination, after which they were cultured into 3D. Two-dimensional and three-dimensional MPM cell blocks were used for protein marker analysis, and our results indicated that the 3D model more closely represents the tumor architecture (Supplementary Figure S1). Protein levels were compared between MPM tumor samples and subsequent derivative cell lines from the same tissue. The detailed percentage scoring of protein marker expression in MPM tumor samples and cell lines is listed in Table 1 (each MM ID represents samples from one patient). It was found that the majority of IHC protein markers are not correlative between tumor tissue and their derived cells (Table 1). Due to the observed variability of protein markers between tissue and cell lines, other molecular biomarker strategies were considered.

### 2.2. Genomic Deletion of CDKN2A Was Identified in MPM Samples Using FISH

**Figure 2:**
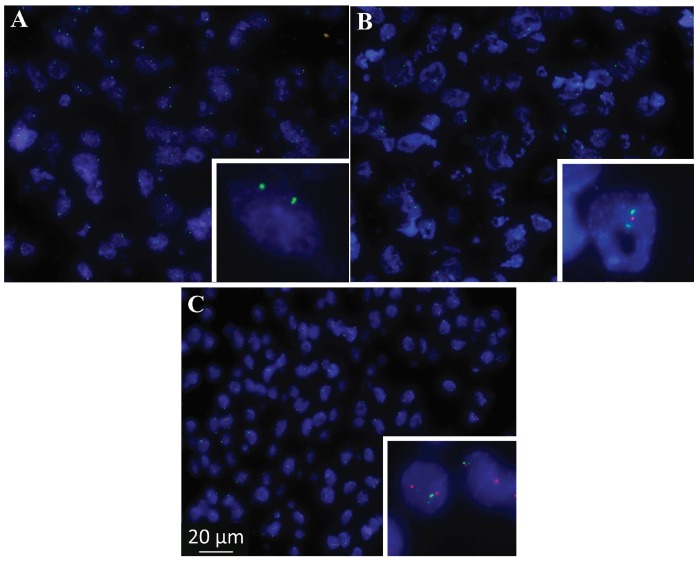
Representative example of Fluorescence In situ Hybridization (FISH) to visualise method of *CDKN2A* deletion in MPM samples. Images depict (**A**) homozygous, (**B**) heterozygous and (**C**) no loss of *CDKN2A* in MPM. The bottom-right corner of each image shows *CDNK2A* (red) as well as control *CEP9* (green) signals. Images were taken using ZEISS Axio Imager M2. All pictures taken at same magnification with scale bar indicated at panel C.

In this study, we have established FISH analysis to identify the genomic deletion of *CDKN2A* using specific probes in MPM tissue samples. FISH staining identified heterozygous or homozygous loss of the *CDNK2A* region in MPM tumor samples (Figure 2); alternatively, normal cells retained expression of both alleles. Figure 2 demonstrates the homozygous loss in the majority of tumor cells (Figure 2A), whereas a smaller portion of samples displayed heterozygous loss (Figure 2B) or no loss (Figure 2C). Of the 12 MPM tumor samples analyzed, 66% (8/12, cut-off 15%) showed homozygous and 16% (2/12, cut-off 40%) showed heterozygous loss of *CDKN2A*. These data indicate that FISH provides a qualitative presentation of genomic deletion in MPM. Although FISH is a well-established technique for the identification of genomic deletion, it is not largely accessible in every laboratory and difficult to provide quantitative assessment; therefore, a more accessible approach to identify genomic changes would be beneficial. To be able to quantitatively analyze genomic deletion, we have assessed the absolute quantification of genomic expression using ddPCR.

### 2.3. Copy Number Variation Contributes to Loss of BAP1 and CDKN2A Expression in MPM

**Figure 3:**
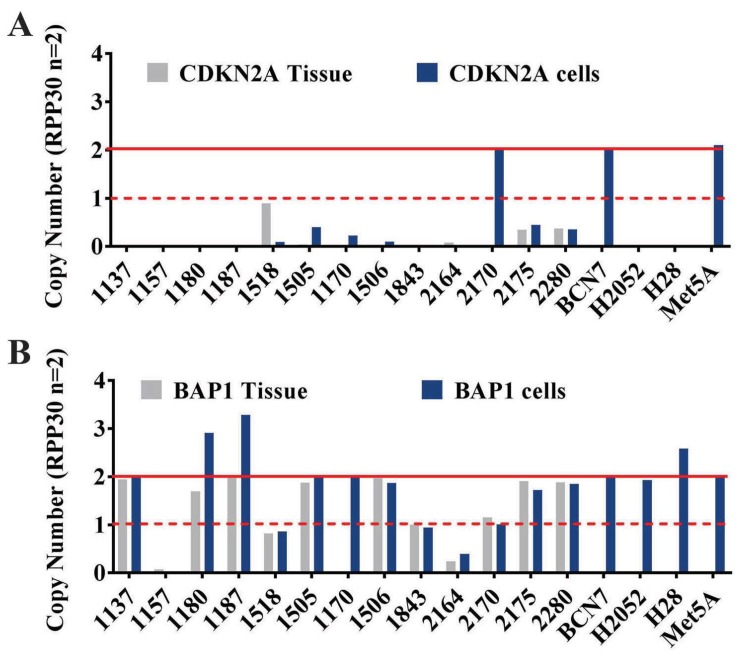
*CDKN2A* and BAP1 protein loss is due to copy number. Copy number (Blue = results from cell line, grey = results from tumor tissue, red line = two copies, red dotter line = one copy) of (**A**) *CDKN2A* and (**B**) *BAP1* in MPM tumor and its derivative cells normalized to the ribonuclease P protein subunit p30 (*RPP30*) assessed by droplet digital PCR (ddPCR).

Our initial attempt to study the DNA content of MPM cells was carried out using metaphase spread and flow-cytometry analysis of DNA content (Supplementary Figure S2). Results indicated cell lines 1180, 1843, 2164, 1157, and 1518 showed tetraploidy. However, these data did not provide conclusive information in regard to specific genes containing copy number variation (CNV). Prior to this study, we reviewed that about 50% MPM cases tested show loss of BAP1 protein expression. Many studies have reported that loss of BAP1 protein expression is due to genetic mutation or DNA methylation in the genomic region. To better understand the mechanism causing *BAP1* loss in MPM, we performed genomic Sanger sequencing of the genetic regions spanning exon 6 and 7, where the majority of mutations reside, as reported in the literature [28,29]. In addition, DNA methylation status was determined by methylation-specific PCR (MSP) analysis to study the involvement of DNA methylation in *BAP1* loss. Results from DNA sequencing and MSP studies (Supplementary Figure S3) indicated no evidence of genomic mutation near exon 6 and 7, and there was no promoter hypermethylation in the nine samples tested. We therefore performed CNV to assess loss of heterozygosity (LOH) of *BAP1* using ddPCR. Results obtained from ddPCR analysis confirm *BAP1* deletion (Figure 3) in MPM samples that correlate with the loss of *BAP1* protein expression observed using IHC analysis (Figure 1). Loss of *CDKN2A* is a common event in mesothelioma [30]. We tested its genomic alteration using FISH and, similar to previous studies, we showed that either heterozygous or homozygous loss of *CDKN2A* is prevalent in our MPM cohort. To assess the potential of detecting copy number loss of *CDKN2A* in MPM samples using ddPCR, we performed CNV ddPCR analysis in MPM tissues and their matched primary cell lines. The percentage of *CDKN2A* loss assessed using ddPCR correlated to results observed using FISH ananlysis (Figure 2). Our results also showed the stability and consistency of CNV detection across formalin-fixed paraffin-embedded (FFPE) samples and established cell lines. Using a normal mesothelial cell line and healthy individual buffy coat (BCN7) samples as normal controls for the presence of both *CDKN2A* alleles and MPM cell lines (H2052 and H28) as controls for gene deletion. Seven percent (one out of 14) of samples showed the retention of both alleles, and the majority of cases (93%: 13 out of 14) showed deletion of the *CDKN2A* genomic region.

### 2.4. Concordance of BAP1 Protein Expression and Genomic Deletion

Table 2 demonstrates BAP1 protein expression and the corresponding ddPCR CNV analysis in matched cell line and tissue samples. In our sample cohort, 54% of MPM FFPE samples showed high levels of BAP1 protein expression, and 46% showed low or no expression of the BAP1 protein. When examining cell lines established from tissue collection (matched samples), 64% showed a high level of expression, while 36% showed low to no expression of BAP1. Strong correlation (92%, 24 out of 26) was demonstrated between the BAP1 protein and genomic expression in FFPE and cell lines samples, as measured by IHC and ddPCR, respectively. Our results indicated low correlation between BAP1 protein and genomic expression, as determined by IHC and CNV. respectively (Supplementary Figure S4). Both FFPE and matched cell-line samples exhibited 50% of *BAP1* genomic retention (13 out of 26) and 70% BAP1 protein expression (19 out of 27), whereas 42% (11 out of 26) of samples that displayed low BAP1 protein expression also showed genomic deletion in the *BAP1* region. SPSS software was utilized for the measurement of sensitivity and specificity of biomarkers for detection of MPM. *CDKN2A* genomic loss had 96.4% of sensitivity and 100% specificity for MPM detection. BAP1 protein expression (using IHC) and genomic deletion (using ddPCR) results were analyzed for their concordance by measuring their combined sensitivity and specificity. Results from SPSS indicated there was 93.3% sensitivity and 63.6% specificity when combining the protein expression and genomic deletion of *BAP1* as a marker for MPM. When analyzing genomic deletion (ddPCR results) alone, *BAP1* genomic loss had a sensitivity of 42.9% and specificity of 100% in identifying MPM compared to normal mesothelial cells and healthy donor buffy-coat controls. In comparison, BAP1 protein expression identifies MPM with lower sensitivity and specificity levels of 67.9% and 28.6%, respectively. These results indicate that detection of *BAP1* and *CDKN2A* by genomic analysis (CNV using ddPCR) is a more distinctive method to identify MPM.

## 3. Discussion

MPM tumors are histologically heterogeneous and have distinct morphological subtypes that range from epithelioid, sarcomatoid, and biphasic. The biphasic histological subtype consists of epithelioid and sarcomatoid components, with each contributing to at least 10% of the tumor. Further, histomorphological features such as mitotic numbers and nuclear atypia have been included to conclude a total score [31]. This heterogeneity within morphological subgroups further adds to the complexity of the definitive identification of MPM [32]. It is therefore important to establish cultured cells from mesothelioma biopsies and pleural effusions to be utilized for studying the cellular, molecular, and genetic levels of this tumor. Over a period of four years (2013–2017), we established fifteen cell lines (defined as successful subculture) from a primary culture [27] from fourteen human tumor and/or plural effusion samples. In the current study, we utilized these established mesothelioma cell lines to determine biomarker expression in a system that closely parallels the tumor.

Currently, no single immunohistochemical marker offers high specificity and sensitivity or definitive negative predictive value for the diagnosis of mesothelioma. The most useful mesothelial and epithelial markers proposed for the diagnosis of mesothelioma are a combination of markers, often including calretinin (a vitamin D-dependent calcium-binding protein involved in calcium signalling) [33], HBME-1, thrombomodulin, WT-1, mesothelin, and podoplanin as mesothelial markers, and pCEA, Ber-EP4, TTF-1, and TAG72 as epithelial markers [34]. The cytokeratin-19/CEA ratio is a useful marker for mesothelioma diagnosis due to the high level of cytokeratin (18 and 19) expression in mesothelial cells. Additionally, cytokeratin-19 was previously found in two hereditary cases of mesothelioma [35]. The IHC panel included a total of 15 markers, and these were tested in the matched tumor and primary cell-line sample set. Unexpectedly, we did not observe a complimentary pattern in expression between tumor samples and derivative cell lines; often, the cell-line marker expression scores deviated from what was observed in the tumor samples. This finding suggests that in terms of MPM identification, IHC marker subtyping is not an ideal method for use in cell lines. Cell-to-cell communication in tumors creates distinct protein expression phenotypes that differ from those in cell culture [36] and this could explain the observed difference in protein marker expression. Analysis of the genomic phenotype could, instead, provide an alternative method of tumor identification.

Homozygous deletion (HD) of *CDKN2A* is one of the most common gene alterations associated with MPM [30]. Detection of *CDKN2A* HD using FISH can be used to differentiate between MPM and RMH (43% to 93% sensitivity; 100% specificity) [37]. However, the *CDKN2A* FISH assay only provides qualitative measurement of genomic expression and is inaccessible to many laboratories due to high costs and a highly specialised workflow. Furthermore, the complex structural chromosomal instability in mesothelioma cell lines can lead to the complication of karyotype differentiation and problems in chromosomal abnormality detection that may not be revealed by routine G-banding or FISH techniques [38]. ddPCR offers an alternative method for genomic analysis that provides absolute quantification, thereby enabling CNV analysis. Additionally, it is relatively cost-effective for routine use [39], thus enabling high-throughput application in the clinic. Hida et al. previously carried out BAP1 IHC analysis and *CDKN2A*-specific FISH in 40 MPM and 20 reactive mesothelial hyperplasia (RMH) samples [40]. Results indicated that BAP1 expression loss and *CDKN2A* homozygous deletion were present in 27 (67.5%) and 17 (42.5%) MPM cases, respectively. Three MPM cases (7.5%) and all 20 RMH cases had neither *BAP1* loss nor *CDKN2A* homozygous deletion. The combination of two markers produced higher sensitivity (92.5%, 37/40) and estimated probability than BAP1 IHC and *CDKN2A* FISH used alone. In our study, the combination of the two markers produced 96.4% of sensitivity and 100% specificity. Our results indicate that CNV analysis of tumor and matched cell-line samples were concurrent and both indicated *CDKN2A* deletion. *BAP1* loss in MPM is attributed to multiple mechanisms including mutation, DNA methylation, or copy number loss [41]. These data show that BAP1 protein loss was due to genomic deletion and *BAP1* CNV at the tumor level was also found to be reiterated in the matched MPM cell lines.

The discovery of alternative molecular markers for MPM is required to facilitate effective diagnosis to improve the dire prognosis of the disease. This study suggests that the CNV of *CDKN2A* is identifiable in MPM tumor samples and derivative cell lines alike using ddPCR. Additionally, *BAP1* CNV was demonstrated using ddPCR and was correlated between tumor and cell-line samples. This highlights the stability of *CDKN2A* and *BAP1* genomic deletion in MPM tumors and suggests identification of CNV could offer a potential alternative in MPM diagnostic testing.

## 4. Materials and Methods

### 4.1. Patient Tissue-Sample Collection and MPM Cell-Line Establishment

#### MPM Cell-Line Establishment

Formalin-fixed paraffin-embedded (FFPE) tumor tissues and fresh tumor samples were collected from MPM patients. All patients gave informed written consent and the project was approved by the Human Research Ethics Committees at Concord Repatriation General Hospital (HREC/11/CRGH/75 approved since 2011). Patient demographics are listed in Table 3. 

### 4.2. Immunohistochemical Analysis of MPM Tissue Sections and Established Cell-Line Blocks

MPM cell lines were cultured into a 3D model and embedded into cell blocks that were further processed into paraffin blocks. MPM tissue blocks and cell blocks were sectioned at 0.4 μm thickness, deparaffinised, and rehydrated in graded concentrations of xylene and ethanol. Antigen retrieval and immunohistochemical staining were performed on an automated Leica Bond III (Leica Microsystems, Melbourne, Australia) using a Bond Polymer Refine Detection Kit (Leica Biosystems, Milton Keynes, UK). Either enzyme 1 (Leica Biosystems, UK) or Heat-Induced Epitope Retrieval (HIER) was performed on all slides in either Bond Epitope Retrieval Solution (Leica Biosystems, UK) 1 (pH6) or 2 (pH9) for 20 min. Primary antibody was applied and incubated for 20 min at room temperature (Table 4). Slides were then immersed in H_2_O_2_ for 5 min to quench endogenous peroxidases. Slides were processed for postprimary detection for 15 min, followed by a polymer for 15 min. 3,3′-diaminobenzidine (DAB with enhancer) chromogenic detection and haematoxylin counterstaining were used. Diagnostic clinical procedures related to diagnosis of the cases were performed in a NATA-approved laboratory using external quality-assurance program (QAP)-validated tests. The method of scoring for each antibody in each case was as per usual clinical diagnostic practice. A negative staining pattern was defined as no staining. Positive staining cells were defined as 1+ (weak), 2+ (moderate), or 3+ (strong) staining intensity in the cells, and the number of cells showing the relevant positive intensity were scored as a percentage over the total number of cells present.

### 4.3. FISH Analysis of CDKN2A Genomic Analysis

FISH dual-color analysis was performed with a *CEP9* Spectrum Green-labelled probe and a Spectrum Orange-labelled, locus-specific *CDKN2A* (p16) probe (Cat. 05J51-001, Abbott Molecular, Sydney, Australia). Briefly, paraffin sections were deparaffinised, dehydrated in ethanol, and washed 3 times with H_2_O. Sections were digested with protease K (0.5 mg/mL) at 37 °C for 20 min. The slides were washed in SSC twice, dehydrated with ethanol, and air-dried. The probes were denatured for 5 min at 95 °C before hybridization. Slides were hybridized overnight at 37 °C and washed in 0.2 × SSC/NP40 71 °C for 2 min. Nuclei were counterstained with DAPI/antifade (Vysis, Abbott Molecular, Sydney, Australia). Each FISH assay included normal lung-tissue sections as a negative control, and sections of mesothelioma previously identified as carrying p16 deletion as a positive control. Analyses were performed with a fluorescence microscope (Axio M2, ZEISS, Oberkochen, Germany) equipped with filter sets with single- and dual-band exciters for Spectrum Green, Spectrum Orange, and DAPI (UV 360 nm). The histologic areas previously selected on the hematoxylin-eosin-stained sections were identified on the FISH-treated slides. Overlapping cells were excluded and individual, and well-defined cells were analyzed and scored. At least 100 cells were scored for each mesothelioma case. Homozygous deletion was defined as the absence of both red *CDKN2A* signals (9p21) in the presence of at least one green chromosome 9 signal (*CEP9*). A heterozygous deletion was defined as the presence of one 9p21 signal, and two *CEP9* signals. The cut-off values were established by methods previously described [42]. The cut-off value was the mean percentage plus three SD using normal mesothelial cell nuclei. We established a cut-off value of 15% for homozygous deletion and 40% for heterozygous deletion.

### 4.4. CDKN2A and BAP1 Genomic Loss Were Suggested by CNV Using ddPCR

Primers for the amplification of the genomic region of *BAP1*, *CDKN2A,* and *RPP30* were optimized using ddPCR EvaGreen (Bio-Rad, California, CA, USA) according to the manufacturer’s recommendations. Eighty nanograms of total genomic DNA was isolated from MPM tissues and MPM cell lines for use as a template for ddPCR. ddPCR reaction mixtures were assembled using 2× EvaGreen ddPCR Supermix (Bio-Rad) and primers at a final concentration of 0.2 μM in a total reaction volume of 20 μL. Reactions were dispensed into each well of droplet generator DG8 cartridge (Bio-Rad). Seventy microliters of Evagreen specific droplet generation oil (Bio-Rad) was used to generate approximately 15,000 to 20,000 droplets using the droplet generator (Bio-Rad). A 40 µL droplet emulsion was then loaded onto a 96-well PCR plate (Bio-Rad). The plate was then heat-sealed with a pierceable foil in the PX1 PCR Plate Sealer (Bio-Rad), and placed in the thermocycler (Bio-Rad T1000). Optimal thermal-cycling conditions were used: 95 °C for 5 min; 35 cycles of 95 °C for 30 s, 60 °C for 30 s, 72 °C for 1 min; and a final step at 72 °C for 1 min. The reaction mixtures were then held at 4 °C until needed. The cycled droplets were read individually with the QX200 droplet reader (Bio-Rad), and analyzed with QuantaSoft droplet-reader software, version 1.7 (Bio-Rad). The error reported for a single well was the Poisson 95% confidence interval. No template controls (NTC) were used to monitor contaminations and primer–dimer formation and determination of the cut-off threshold (Supplementary Figure S5), copy number for each genomic region was calculated by normalization to the included reference gene *RPP30* (retains two copies per cell). Homozygous deletion was considered in cases where no detection of the target genomic region was determined, but a distinctive *RPP30* population was apparent. MPM tissue samples and matched cell-line positive populations were used to calculate the positive expression values, and results were plotted as copy number detected per sample.

### 4.5. Statistics

All statistical analyses were carried out using IBM SPSS software version 25. The sensitivity and specificity of *BAP1* and *CDKN2A* deletion were performed using the crosstabs function of in the descriptive statistics of SPSS.

## 5. Conclusions

Loss of *BAP1* and *CDKN2A* are important diagnostic biomarkers in MPM. This study demonstrated the feasibility of genomic deletion as an appropriate biomarker for MPM detection that is consistent in both MPM tumor tissue and matched MPM cell lines.

## Figures and Tables

**Table 1:** Mesothelioma biomarker scoring.

MM ID	Sample Type	CK 8/18	Calretinin	CK 5/6	CD141	HBME-1	WT-1	D2-40	EMA	CEA	TAG72	BG8	CD15	TTF-1	BAP1	Ber-EP4
**1137**	Tissue	(+++) 90%	-	(+++) <5%	(++) 10%	-	(++) 10%	(+) <5%	-	-	-	(++) 70%	-	-	(+++) 90–100%	-
**1137 T**	3D cells	(+++) 100%	-	-	-	-	-	-	-	-	-	(++/+++) 90–100%	-	-	(+++) 30%	-
	2D cells	(+++) 90%	(++) 5%	(+) 10%	(+) 20%	(+++) 10%	(+) 10%	-	(+) <5%	(++) 10%	-	(++) 60%	-	(+++) <5%	-	(++/+++) 60%
**1157**	tissue	(+++) 90–100%	(+++) 80%	(+++) 90–100%	(+) 50%	(+++) 10%	(++) 90%	(++) 30%	(+++) 40%	-	-	(+++) 90–100%	-	-	-	-
**1157 T**	3D cells	(+++) 100%	(+) 40%	(+++) 70%	-	-	(+++) 90%	(+) <10%	(+++) 40%	-	-	(+) 40%	-	-	-	-
	2D cells	(+++) 90–100%	-	(++/+++) 90%	(+) <5%	-	-	-	(+) <5%	-	-	-	-	-	-	(+) 50%
**1180**	Tissue	(++++) 90–100%	(+++) 30%	(+++) <5%	(+++) 30%	(+++) <5%	-	(+++) 10%	-	-	-	(++) 80%	-	-	(+++) 80%	-
**1180 T**	3D cells	(+++) 100%	-	-	-	-	(++) 30%	-	(++) 40%	-	-	(+) 30%	-	(+) 10%	(+++) 95%	-
	2D cells	(+++) 90–100%	-	-	-	-	(+++) <10%	-	-	-	-	-	-	(++) <10%	(+++) 95%	-
**1187**	tissue	(+++) 90–100%	(+++) 90–100%	(+++) 90–100%	(+++) 90–100%	(+++) 90–100%	(+++) 90–100%	(+++) 40%	-	-	(+++) 90–100%	-	-	(++) 90–100%	-	(++) 90–100%
**1187 T**	3D cells	(++) 10%	-	-	-	-	(+++) 90%	(++) 40%	-	-	-	-	-	-	(+++) 80%	-
	2D cells	(+++) 100%	-	-	(++) 40%	-	(++) 80%	(+) 10%	-	-	-	(+) 70%	-	-	(+++) 95%	(+) 80%
**1505**	Tissue	(+++) 90–100%	(+++) 40%	(+++) 10%	(++) 30%	-	(++) <5%	(+++) <5%	-	-	-	(+++) 90–100%	-	(+++) 40%	(+++) 90–100%	-
**1505 T**	3D cells	(+++) 100%	(+) 20%	(+) <5%	(+) <5%	(+) <5%	(+) <5%	-	(++) 60%	(++) 60%	(+) 20%	(+++) 100%	-	(+++) 80%	(+++) 100%	(+++) 50%
	2D cells	(+++) 100%	(++) 80%	(+) 80%	(+) 70%	(+) 20%	-	(+) 30%	-	(+) <5%	(++) 60%	(+++) 100%	(+) 80%	(+++) 80%	(+++) 100%	(+++) 100%
**1506**	Tissue	(+++) 90%	(+++) 80%	-	(++) 70%	(+++) 100%	(++) 60%	(+) 10%	-	-	-	(++) 40%	-	-	(+++) 90%	(+) 10%
**1506 T**	3D cells	-	-	(+) <5%	(+++) 40%	-	-	-	(++) 80%	-	-	(+++) 90%	-	-	(+++) 100%	(+++) 80%
	2D cells	(+) 10%	-	(+) 40%	(+) 10%	(+) <5%	-	-	(++) 40%	(+) 20%	(++) 70%	(+++) 100%	(+) 90%	(+) <5%	(+++) 80%	(+++) 100%
**1518**	Tissue	(+++) 40%	-	(+++) 30%	(+) 20%	-	(++) 10%	(++) 30%	(++) 10%	-	-	(+) 40%	-	-	(+++) 20%	-
**1518 P**	3D cells	(+++) 100%	(+) 10%	(+) <5%	-	(+) 5%	(+++) 40%	(+) <5%	(+) <5%	-	-	(+++) 100%	-	-	(+++) 90%	(+++) 90%
	2D cells	(+++) 100%	(+) 5%	-	(+) 10%	-	(+) 10%	(+) <5%	(+) <5%	(+) 10%	-	(+++) 100%	(+) 80%		(++) 80%	(+++) 100%
**1518 T**	3D cells	(+++) 70%	(++) 10%	-	(+) <5%	-	-	-	-	-	-	-	-	-	-	(+) 10%
	2D cells	-	(++) 4%	-	(+) <5%	-	(+++) 40%	(++) 30%	-	(++/+++) 10%	(+) 10%	-	-	(+++) 20%	-	(+++) 40%
**1170 T**	3D cells	(++/+++) 70%	-	-	(+) 40%	(++) <5%	(+++) 20%	-	(+++) 30%	-	-	(+) 10%	(+) <5%	(+++) 90–100%	(+++) 95%	(++/+++) 40%
	2D cells	(++) 70%	-	-	-	(++) 70%	(++/+++) 80%	-	-	-	-	-	-	(+++) 80%	(+++) 95%	(++) 20%
**1843**	Tissue	(++) 40%	(+) <10%	(+++) 40%	(+++) 30%	(+++) 30%	(+) 5%	-	(+++) 20%	-	-	(+) 20%	-	(+) <5%	-	-
**1843 T**	3D cells	(+++) 50%	-	-	-	-	(+++) 100%	(+) 20%	(++/+++) 40%	-	-	-	-	(+++) 90%	-	-
	2D cells	(++) 80%	-	-	-	-	(++) 70%	-	-	-	-	(+) 50%	-	(++) 80%	-	(+) 50%
**2164**	Tissue	(+++) 40%	(++) 40%	-	(++) 40%	-	(+) <5%	(+++) 70%	(+++) 60%	-	-	(+) 10%	-	-	-	-
**2164 P**	3D cells	-	-	-	-	-	(++) 90%	-	-	-	-	-	-	-	-	-
	2D cells	-	-	-	-	-	(++) 90–100%	(++) 30%	(+) 10%	-	-	(+) 40%	-	-	-	-
**2170**	Tissue	(+++) 90%	(+++) 60%	(+) 5%	(++) 40%	(+++) 90%	(+++) 40%	(+++) 80%	(+++) 80%	-	-	(++) 10%	-	-	-	(+++) 5%
**2170 T**	3D cells	-	(+) <5%	-	-	-	-	-	-	(+) 15%	-	(+++) 100%	(++) 5%	-	(+++) 100%	-
	2D cells	-	-	-	(++) 10%	(+) 5%	-	(+) <5%	(+) 10%	-	-	(+++) 100%	(+) 10%	-	(+++) 100%	(+) 80%
**2174 P**	3D	(+++) 100%	(++) <5%	-	(+++) 50%	(+++) 100%	(+++) 80%	-	(++) 40%	-	-	(+++) 100%	-	-	(+++) 100%	-
	2D	(+++) 80%	(++) 80%	-	(+++) 90%	(+) 20%	-	(+++) 90%	(+++) 80%	(+) 10%	-	(+++) 100%	(+) 90%	-	(+++) 70%	(+) 90%
**2175**	Tissue	(+++) 100%	(+++) 60%	(+++80%)	-	(+) 10%	(+) 20%	(+) 50%	(+++) 70%	-	-	-	-	-	(+++) 80%	-
**2175 P**	3D cells	-	-	-	(+++) 60%	-	-	-	(++/+++) 80%	-	-	-	-	-	(+++) 95%	-
	2D cells	-	-	-	(++) <5%	-	-	-	(+) <5%	-	-	-	-	-	(+++) 95%	-
**2280**	Tissue	(+++) 100%	(+) <5%	-	(+++) 90%	-	-	-	(+) <5%	-	-	-	-	-	(+) 100%	-
**2280 T**	3D cells	-	-	-	(+++) 60%	-	-	-	(+++) 80%	-	-	(+++) 80%	-	-	(+++) 100%	-
	2D cells	(+++) <5%	-	-	(+) 5%	-	-	(+) <5%	(+) 10%	-	-	(+++) 100%	(+) 20%	-	(+++) 100%	(+) 90%

T = cell lines established from MPM tumor tissue; P = cell lines established from MPM pleural effusion. +, ++, +++ = 1 positive, 2, positive, 3 positive of IHC intensity.

**Table 2:** Concordance of BAP1 IHC and ddPCR analysis.

MM ID	Sample Type	BAP1 IHC	BAP1 ddPCR (Reference to RPP30)
**1137**	Tissue	(+++) 90–100%	1.94
	cells	(+++) 30%	2
**1157**	Tissue	ND	0.028
	cells	ND	0.07
**1180**	Tissue	(+++) 80%	2.91
	cells	(+++) 95%	1.69
**1187**	Tissue	ND	3.28
	cells	(+++) 80%	2
**1505**	Tissue	(+++) 90–100%	1.87
	cells	(+++) 100%	2.01
**1506**	Tissue	(+++) 90%	1.96
	cells	(+++) 100%	1.87
**1518**	Tissue	(+++) 20%	0.82
	cells	(+++) 90%	0.86
**1170**	Tissue	(+++) 95%	No tissue availible
	Cells	(+++) 95%	1.98
**1843**	Tissue	ND	1
	Cells	ND	0.94
**2164**	Tissue	ND	0.24
	Cells	ND	0.39
**2170**	Tissue	ND	1.15
	Cells	(+++) 100%	1
**2174**	Tissue	No tissue availible	No tissue availible
	Cells	(+++) 70%	1.327814
**2175**	Tissue	(+++) 80%	1.91
	Cells	(+++) 95%	1.92
**2280**	Tissue	(+) 100%	1.88
	Cells	(+++) 100%	1.85

ND = not detected. +, ++, +++ = 1 positive, 2, positive, 3 positive of IHC intensity.

**Table 3:** Patient demographics.

MM ID	Aga at Diagnose	Gender	Histological Subtype	Surgery Procedure	Survival (months)	Asbestos Exposure
1137	83	Male	Desmoplastic	Biopsy, Decortication, Pleurodesis	0.7	Yes
1157	51	Male	Epitheliod	Biopsy, Decortication, Pleurodesis	20.6	Yes
1170	74	Male	Biphasic	Biopsy, Decortication, Pleurodesis	0.8	Yes
1180	72	Male	Biphasic	Biopsy, Decortication, Pleurodesis	7.4	Yes
1187	64	Male	Biphasic	Extrapleural pneumonectomy	33.5	Yes
1505	57	Male	Epitheliod	Biopsy, Surgical exploration	7.2	Yes
1506	72	Male	Epitheliod	Biopsy, Pleurodesis	24.0	Yes
1518	80	Male	Biphasic	Biopsy, Decortication, Pleurodesis	22.7	Yes
1843	60	Male	Biphasic	Decortication, Pleurodesis	15.6	Yes
2164	73	Male	Epitheliod	Biopsy, Decortication, Pleurodesis	*	Yes
2170	75	Male	Epitheliod	Biopsy, Decortication, Pleurodesis	*	ND
2174	78	Male	Epitheliod	Biopsy, Decortication, Pleurodesis	3.0	Yes
2175	66	Male	Epitheliod	Biopsy, Decortication, Pleurodesis	12.5	Yes
2280	69	Male	Epitheliod	Extrapleural pneumonectomy	*	Yes

* = still alive; ND = no data provided.

**Table 4:** Antibodies used in this study.

Antibody	Clone	Manufacturer	Product Code	Species	Dilution
CK8/18	EP17/30	Dako	M3652	Rabbit	1:100
Calretinin	Polyclonal	Biocare	CP092C	Rabbit	1:100
CK5/6	D5 & 16B4	Cell Marque	358M-16	Mouse	1:150
CD141	15CB	Novocastra	NCL-CD141	Mouse	1:50
HBME1	HBME-1	Dako	M3505	Mouse	1:50
WT1	WT49	Novocastra	NCL-L-WT1-562	Mouse	1:50
D2-40	D2-40	Biocare	CM266C	Mouse	1:100
EMA	GP1.4	Novocastra	NCL-L-EMA	Mouse	1:350
CEA	11-7	Dako	M7072	Mouse	1:200
TAG72	B72-3	Cell Marque	337M-84	Mouse	1:2000
BG8	F3	Covance	SIG-3317-1000	Mouse	1:100
CD15	Carb-3	Dako	M3631	Mouse	1:100
TTF-1	SPT24	Novocastra	NCL-L-TTF-1	Mouse	1:100
BAP1	C-4	Santa Cruz	SC-28383	Mouse	1:200
HEA	Ber-EP4	Dako	M0804	Mouse	1:100

## Supplementary Materials

Supplementary materials can be found at http://www.mdpi.com/1422-0067/19/10/3056/s1.
