# Supplementary material for: Genomic Deletion of BAP1 and CDKN2A Are Useful Markers for Quality Control of Malignant Pleural Mesothelioma (MPM) Primary Cultures
Source: Int J Mol Sci. 2018 Oct 7;19(10):3056. doi: 10.3390/ijms19103056 (PMC6213505; doi:10.3390/ijms19103056)
Supplement: Supplementary file 1 [file ijms-19-03056-s001.pdf]

Supplementary

Methods

Culturing of Primary Cells from Patient Pleural Samples

Patient pleural tumor and effusion samples were processed before attempting to grow in tissue culture. Pleural tumor samples were received in RPMI 1640 medium (Life Technologies) supplemented with 10% fetal bovine serum (Life Technologies, Carlsbad, CA, USA) and 10% penicillin-streptomycin (Life Technologies, Carlsbad, CA, USA). This medium was aspirated and replaced by 5 mL of fresh medium of the same components and incubated under normal tissue-culture conditions for 10 min. The medium was aspirated once more before the tumor sample was placed in a petri dish and cut into small pieces 1–3 mm in diameter using a sterile scalpel. The cut tumor samples were then incubated in RPMI 1640 medium supplemented with collagenase type I (Life Technologies) at 200 U/mL concentration for 10 min under normal tissue-culture condition to digest collagen and release tumor cells. The tumor sample suspension was centrifuged and supernatant removed. Tissue samples were then resuspended in 2.5 mL of RPMI 1640 supplemented with 10% fetal bovine serum and 1% penicillin-streptomycin. This suspension was transferred to a T25 cm^2 tissue-culture flask ensuring that tumor sample pieces were in contact with the tissue culture-treated bottom of the flask. The flask was then incubated under normal tissue-culture conditions and left undisturbed for at least 3 days. This allowed for cells to disperse from the tumor samples and attach onto the T25 cm^2 tissue-culture flask. Once the T25 cm^2 flask was confluent, cells were transferred to a larger T75 cm^2 flask and cultured for at least 15 passages to ensure stable growth of tumor cells. For pleural effusion, samples were centrifuged to obtain a cell pellet and supernatant was aspirated. To remove red-blood-cell contamination, the cell pellet was resuspended in red-blood-cell lysis buffer, centrifuged, and aspirated multiple times until the solution was clear and cell pellet was white. The clean cell pellet was then finally resuspended in 10 mL of RPMI 1640 medium supplemented with 10% fetal bovine serum and 1% penicillin-streptomycin and transferred into a T75 cm2 tissue-culture flask. The cells were then cultured for at least 15 passages to ensure stable growth of tumor cells.

Ploidy Analysis

To assess the extent of chromosomal DNA alteration in primary MPM cells, ploidy analysis was performed using DNA binding propidium iodide. Primary MPM cell lines, including normal fibroblast cells, were seeded in a 10 cm tissue culture plate at a density of 1×10^6 cells/plate. Once about 80% confluent, cells were harvested and washed 3 times with PBS (Life Technologies, Carlsbad, CA, USA). Cells were fixed overnight in 70% ethanol at 4 °C. Fixing solution was removed and cells were treated with 12.5 µg/mL of RNase A (Sigma, St. Louis, MO, USA) and 15 µg/mL of propidium iodide (Sigma, St. Louis, MO, USA) in PBS for 30 min at 37 °C in the dark. The extent of propidium iodide staining, hence DNA content was measured by an Accuri C6 flow cytometer (BD Biosciences, Franklin Lakes, NJ, USA). The flow cytometer was calibrated using 6- and 8-peak fluorescent bead mixtures provided by the manufacturer, and according to the manufacturer's instructions (BD Biosciences, Franklin Lakes, NJ, USA). The flow cytometer was operated at the Slow Flow Rate setting of 14 µL sample/minute, and gated to detect 1×10^4 single cell events. The data was exported to and analysed on the FlowJo software. The results of primary MPM cells were compared against normal fibroblast cells which possess a diploid ($2n$) set of chromosomal DNA.

Metaphase Spreads

To visualize changes in chromosomal DNA in primary MPM cells, metaphase spreads were generated and DNA stained using DAPI. Primary MPM cell lines, including normal fibroblast cells, were seeded in a 10 cm tissue-culture plate at a density of 1×10^6 cells/plate. After 48 h, cells were treated with a final concentration of 100 ng/mL of KaryoMAX® Colcemid™ solution (Life Technologies, Carlsbad, CA, USA) in culture medium to arrest dividing cells at metaphase. The cells were then left to incubate for 2 h under tissue-culture conditions. Cells were observed under a light microscope to identify round cells that have undergone arrest at metaphase.

These cells were slightly agitated to detach and harvested with the medium. If more metaphase-arrested cells were needed, a brief wash with PBS and Trypsin-EDTA (Life Technologies, Carlsbad, CA, USA) helped to increase cell numbers without disturbing remaining cells. Harvested cells were centrifuged and supernatant removed, leaving behind about 0.5 mL to gently resuspend cell pellet. Hypotonic KCl (75 mM), prewarmed to 37 °C, was added dropwise while gently vortexing to a total volume of 10 mL. The cells were then incubated at 37 °C for about 10 min until sufficiently swollen, and 2 ml of fresh fixative solution (Methanol: Acetic acid, 3:1) was added to stop the reaction. Swollen cells were again centrifuged and the supernatant removed, leaving behind about 0.5 mL to gently resuspend cell pellet. To fix cells, 10 mL of fresh fixative was added dropwise while gently vortexing. Cells were left to fix for 10 min at room temperature before centrifuging and removing supernatant leaving behind 0.5 mL. The cells were washed in fresh fixative 2 more times before resuspending to an optically opaque cell density. A drop of fixed cells was placed onto a clean slide and tapped to run down and left to dry for about 15 min at room temperature. Cells were then mounted and stained using ProLong® Diamond Antifade Mountant with DAPI (Life Technologies, Carlsbad, CA, USA). Metaphase spread images were then captured on a Zeiss Axio Imager M2 fluorescent microscope using a 63× oil objective.

BAP1 DNA Methylation Analysis Using MSP

Genomic DNA from MPM samples was analysed for its BAP1 promoter methylation status using MSP. Briefly, 2 μg of genomic DNA was bisulfite-treated with the Zymo DNA Modification Kit (Zymo Research, Orange, CA, USA) and resuspended in 50 μL H2O (~40 ng/μL). Bisulfite-treated DNA was used as a template for MSP PCR. PCR was carried out on a MultiGene Gradient thermal cycler (Labnet International Inc., Woodbridge, NJ, USA). PCR products from MSP analysis were then separated in nondenaturing polyacrylaminde gels and photographed after staining with ethidium bromide.

Table S1: Ten loci STR profiling results of culture-established MPM cell lines.

MMID	1137		1157		1170		1180		1187	
Marker	Allele 1	Allele 2	Allele 1	Allele 2	Allele 1	Allele 2	Allele 1	Allele 2	Allele 1	Allele 2
AMEL	X	Y	X	Y	X	ND	X	Y	X	Y
CSF1PO	12	14	10	12	11	11	11	12	11	12
D13S317	11	13	11	12	8	8	11	11	10	10
D16S539	10	11	11	13	11	11	11	12	9	12
D21S11	29	31.2	29	30	30	31	28	32.2	29	29
D5S818	11	11	13	13	11	11	10	11	11	13
D7S820	9	9	10	12	8	8	10	12	8	10
TH01	9.3	9.3	6	6	8	9	8	9.3	6	7
TPOX	8	8	8	8	11	11	8	8	9	11
vWA	16	18	18	19	19	19	14	15	17	18

MM ID	1505		1506		1518		1843		2164	
Marker	Allele 1	Allele 2	Allele 1	Allele 2	Allele 1	Allele 2	Allele 1	Allele 2	Allele 1	Allele 2
AMEL	X	Y	X	Y	X	Y	X	Y	X	Y
CSF1PO	10	12	11	12	10	12	10	11	11	12
D13S317	12	12	11	13	12	14	8	12	12	12
D16S539	11	12	11	13	11	11	11	14	10	11
D21S11	29	32.2	30	31.2	31	31	28	30	30	30
D5S818	12	13	11	12	12	12	9	11	12	13
D7S820	9	12	10	12	9	10	8	12	11	12
TH01	8	9.3	6	9.3	6	9.3	7	9.3	6	9.3
TPOX	8	8	8	11	9	11	8	11	8	9
vWA	15	18	17	18	17	17	15	17	16	16

MM ID	2170		2174		2175		2280	
Marker	Allele 1	Allele 2	Allele 1	Allele 2	Allele 1	Allele 2	Allele 1	Allele 2
AMEL	X	Y	X	ND	X	Y	X	Y
CSF1PO	11	12	10	11	12	13	9	12
D13S317	11	12	8	12	9	11	11	12
D16S539	10	11	13	13	9	12	10	12
D21S11	28	30	28	30	30	33.2	30.2	31.2
D5S818	11	13	9	12	11	13	12	12
D7S820	10	10	9	9	8	10	11	11
TH01	9	9	7	7	6	9	6	9
TPOX	8	11	7	9	8	11	8	10
vWA	17	19	17	17	17	18	16	19

ND = not detected. The Y Allele in patients 1170 and 2174 was not detected. The AMEL (Amelogenin) locus is often the preferred locus for distinguishing between male and female samples; however, there are published instances (PMID: 15726419, 16781100) where a PCR assay at this locus did not differentiate the sex accurately—these occurrences are usually due to biological factors (most commonly large-scale deletion events). A similar report from an established ATCC MPM cell line H2052 (male) also demonstrates the absence of Y allele detection in a male sample.

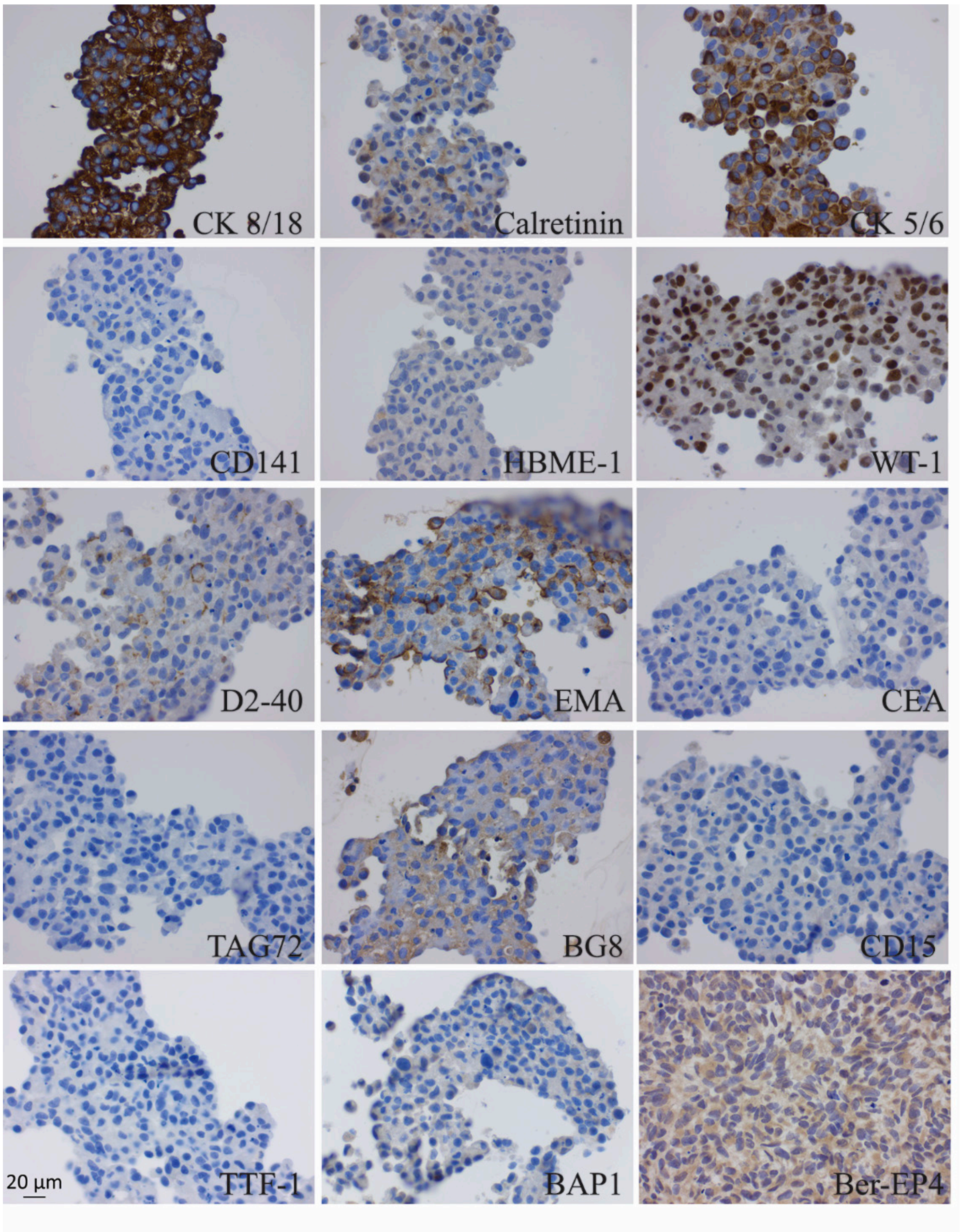

Figure S1. Representative IHC staining (sample ID 1157) of a cultured 3D MPM sample, with the 15 biomarkers currently used for clinical diagnosis for MPM. All pictures taken at same magnification with scale bar indicated left bottom corner of TTF-1 staining.

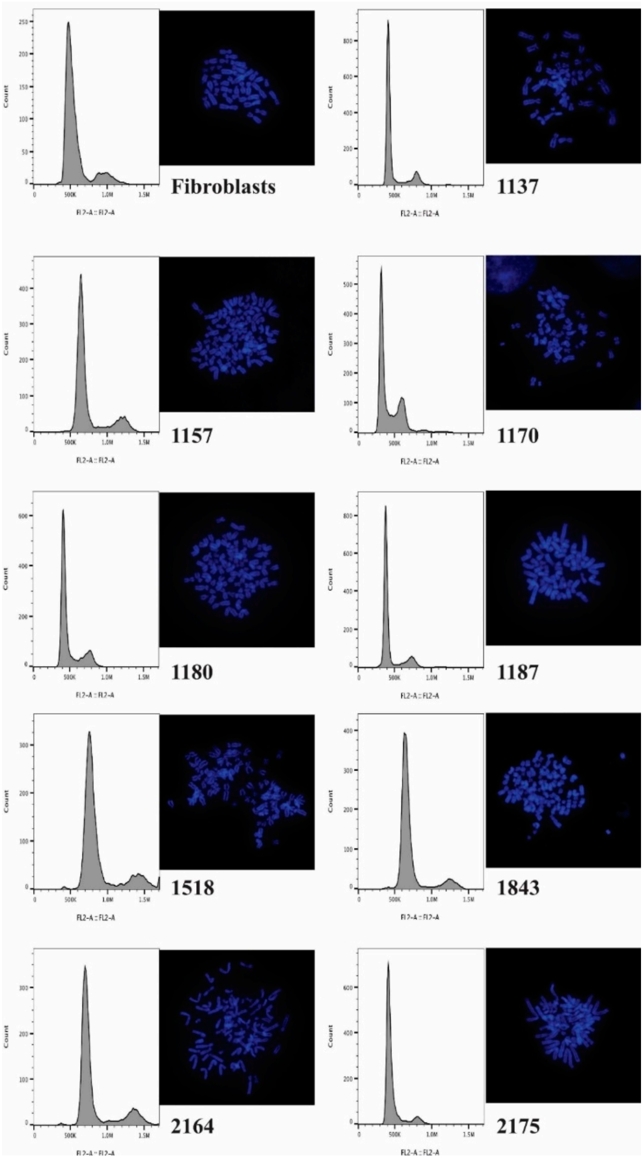

Figure S2. Ploidy and metaphase spread analysis of chromosomal DNA alterations in established MPM cell lines.

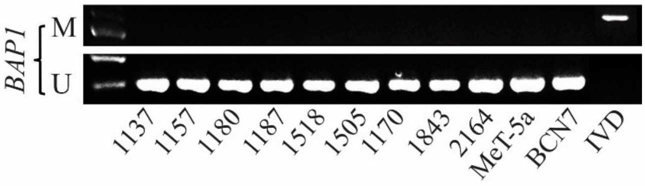

Supplementary Figure 3. *BAP1* MSP result.

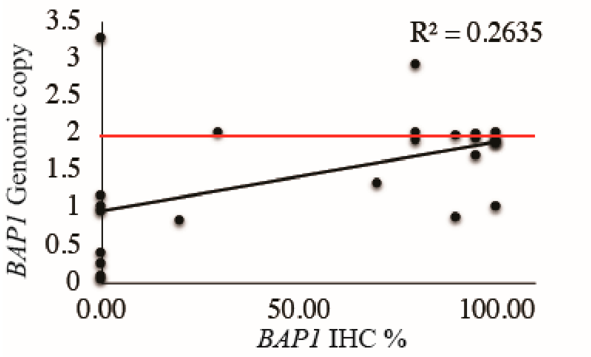

Figure S4. Correlation plot of *BAP1* IHC and copy number variation (CNV).

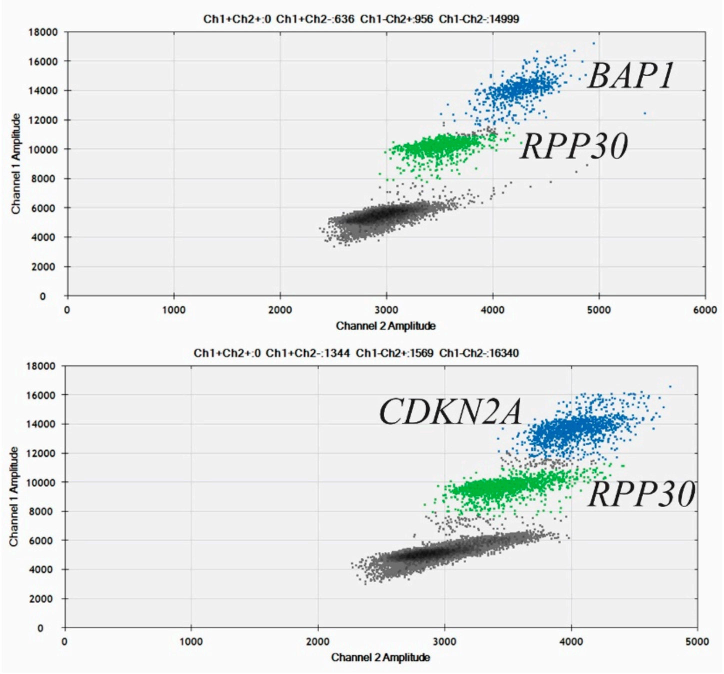

Figure S5. ddPCR generated CNV results for *BAP1* and *CDKN2A* with homozygous RPP30 expression as reference. Healthy normal individual buffy-coat DNA (BCN7) was used for assay optimisation. Distinctive BAP1 and CDKN2A population are detectable with similar read when compared to reference RPP30 gene detection. The following cut-off was used: RPP30 was used as reference of two genomic copies per cell. A copy number below 2 (range 0.8–1.7) represents heterozygous deletion; a copy number below 1 (range 0 to 0.7) represents homozygous deletion.
